# Clinical Implication of Adherent Perinephric Fat in Robot-Assisted Partial Nephrectomy: Validation With Video Review

**DOI:** 10.3389/fsurg.2022.840664

**Published:** 2022-04-08

**Authors:** Hwanik Kim, Myeongju Kim, Seok-Soo Byun, Sung Kyu Hong, Sangchul Lee

**Affiliations:** ^1^Department of Urology, Seoul National University Bundang Hospital, Seongnam, South Korea; ^2^Department of Urology, Hallym University Sacred Heart Hospital, Hallym University College of Medicine, Anyang, South Korea; ^3^Center for Artificial Intelligence in Healthcare, Seoul National University Bundang Hospital, Seongnam, South Korea; ^4^Department of Urology, Seoul National University College of Medicine, Seoul, South Korea

**Keywords:** adherent perinephric fat, renal cell carcinoma, robot-assisted partial nephrectomy, perirenal fat dissection time, MAYO adhesive probability score, toxic perirenal fat

## Abstract

**Objective:**

To assess the impact of adherent perinephric fat (APF) on perioperative outcomes of robot-assisted partial nephrectomy (RAPN).

**Methods:**

A total of 562 Asian patients with kidney tumors received RAPN and their Mayo adhesive probability (MAP) scores were evaluated. APF was determined intraoperatively and confirmed by a second surgical video review and perioperative data were compared according to the MAP score. The associations of APF with clinical factors were examined using logistic regression analyses. Subgroup (classified according to who performed the surgery) analysis was conducted to assess if the perirenal dissection time is significantly correlated with APF.

**Results:**

A total of 118 consecutive patients were classified into two groups according to APF. Patients in the APF group needed significantly longer perirenal fat dissection time (*p* < 0.001) and longer hospital stay (*p* = 0.028). MAP score (Odds ratio [OR]: 2.71, 95% Confidence interval [CI]: 1.56–4.71, *p* < 0.001), body mass index (OR: 1.24, 95% CI: 1.04–1.47, *p* = 0.016), and perirenal fat dissection time (OR: 1.11, 95% CI: 1.03–1.19, *p* = 0.004) were significantly associated with the presence of APF. Perirenal fat dissection time was significantly correlated with APF presence in two of three surgeon subgroups (ß = 8.117, *p* = 0.023; ß = 7.239, *p* = 0.011).

**Conclusions:**

Preoperative MAP score and perirenal fat dissection time were significantly associated with APF during RAPN.

## Introduction

It is not rare to experience hard cases of robot-assisted partial nephrectomy (RAPN) even by expert robotic surgeons. The presence of thick and adherent ‘toxic' perinephric fat during RAPN can handicap the surgical procedure and impair capabilities to secure clear surgical boundaries of a renal tumor at the surface of the kidney. Radiographic features of preoperative perinephric fat based on CT density measurements are predictive of ease of surgical dissection based on perirenal fat adherence. The level of difficulty for surgical dissection during partial nephrectomy (PN) is encompassed by several factors including the location and size of the renal mass and its relationship with renal hilar vessels and the urinary collecting system. Moreover, a significant component of factors affecting the level of difficulty for PN pertains to the quantity and quality of perinephric fat in that given renal unit (1). Mayo adhesive probability (MAP) score is an exact tool for predicting adherent perinephric fat (APF) and has been validated in some Western populations (2–4). In Asian cohorts, the APF presence during RAPN has been scarcely investigated and the predictive capability of the MAP score has not been fully unexplored. Thus, the aim of this study was to investigate the impact of APF on perioperative outcomes of RAPN.

## Methods

The study was conducted in accordance with the Declaration of Helsinki (as revised in 2013). The study was approved by the institutional review board (IRB) of Seoul National University Bundang Hospital (NO.: B-2108-702-102) and individual consent for this retrospective analysis was waived.

### Data Collection

From January 2019 to August 2020, 562 patients who received RAPN by multiple surgeons at a single institution and who were eligible for surgical video review were analyzed. Three surgeons with more than 10 years of experience in robotic surgery and more than 100 RAPN participated in the study. A total of 47, 39, and 32 cases were performed by surgeons A, B, and C, respectively. Patients were excluded from the analysis if the actual peripheral fat dissection time was difficult to measure because the video was not properly recorded or missed. APF was determined intraoperatively by a surgeon at first. Another researcher further conducted a video review. If APF's judgment was inconsistent, the presence of APF was decided by consensus from the review of videos and medical records. Video reviewers were blinded to patient characteristics including the MAP score.

Adherent perinephric fat was defined as a fatty region around the kidney within its anatomical structures, including the fusion fascia, diaphragm, lateroconal fascia, lumbar quadrate muscle, and psoas muscle. APF was determined intraoperatively and confirmed through a second surgical video review by a urologist as perinephric fat within the Gerota's fascia that adhered to the renal parenchyma and was hard to dissect for identification of the kidney tumor (5).

Adherent perinephric fat was considered to be present if at least one of the criteria was met. The criteria for APF or toxic fat were as follows: (1) when adipose tissue was dissected around the kidneys and fat was layered on top of each other; (2) when dissecting the adipose tissue with severe bleeding and meticulous hemostasis were required; (3) when some inseparable adipose tissues adhered to the kidney parenchyma around the tumor and must be removed together with the tumor, although kidney parenchyma was left with most of the fat contents peeled off. APF from our point of view was not related to intestinal adhesions or intestinal adhesions with Gerota's fascia in the abdominal wall or kidney.

The definition of the peripheral fat dissection time was as follows. Once renal arteries and veins were secured for further hilar clamping control (not counted as peripheral fat dissection time), it was defined as the time from commencement of dissection of the adipose tissue around the kidneys to the moment until just before confirming the tumor's delicate location or depth with ultrasound.

### Study Endpoints

Our primary endpoint was a significant association of adherent perinephric fat with perioperative factors. The secondary endpoint was the difference of clinicopathological characteristics between groups classified by APF presence. Subgroup, classified by each surgeon, analysis was conducted to assess if perirenal dissection time is significantly correlated with APF.

### Statistical Analysis

Comparisons between the two groups were assessed using the Chi-squared test for categorical variables and Mann-Whitney U test or independent *t-*test for continuous variables. Univariate and multivariate logistic regression analyses were implemented to evaluate associations of APF with perioperative clinical factors. Multicollinearity among covariates was evaluated using the variance inflation factor before conducting multivariable analysis to minimize confounding bias. Variables with variance inflation factor >5 were excluded from the analysis and all variables in the multivariate analysis were <5 ranging from 1.25 to 1.56. Univariate results were used in the final multivariate model to determine candidate variables through a backward model selection process. For all variables remaining in the last multivariate analysis, the *p*-value was set to.05. All data were analyzed with SPSS version 22 and all tests were two-sided with a *p*-value of.05 considered statistically significant (IBM SPSS Statistics, IBM Corp., Armonk, NY, USA).

## Results

The baseline information, tumor features, and operative outcomes of 118 consecutive patients with RAPN are presented in Table 1. A total of 444 ineligible patients were excluded for analysis due to video of inadequate quality or missing data. None was converted to radical nephrectomy or open technique owing to intraoperative complications or surgical difficulty.

**Table 1:** Perioperative outcomes between no adherent perinephric fat (APF) and APF groups.

**Variable**	**No APF (*n* = 80)**	**APF (*n* = 38)**	***p*** **-value**
Age	48.2 ± 11.7	55.8 ± 14.9	0.003
Body mass index	24.4 ± 3.4	26.9 ± 3.7	<0.001
Gender			0.111
Male / Female	54 (67.5%) / 26 (32.5%)	31 (81.6%) / 7 (18.4)	
ECOG 0 / ≥1	70 (87.5%) / 10 (12.5%)	33 (86.8%) / 5 (13.2%)	0.758
DM	5 (6.3%)	6 (15.8%)	0.096
HTN	23 (28.7%)	19 (50.0%)	0.024
CKD	1 (1.3%)	3 (7.9%)	0.062
Preoperative serum creatinine	0.80 ± 0.20	0.92 ± 0.25	0.004
Preoperative eGFR	100.7 ± 24.9	87.5 ± 23.5	0.007
Tumor size (mm)	32.4 ± 14.7	40.7 ± 18.9	0.339
**Clinical T stage**			0.064
T1a / T1b	56 (70.0%) / 23 (28.7%)	20 (52.6%) / 15 (39.5%)	
T2	1 (1.3%)	3 (7.9%)	
RENAL score	8.0	9.0	0.953
MAP score	1.3 ± 1.2	3.1 ± 1.3	<0.001
Operation time (min)	119.8 ± 39.7	132.1 ± 36.5	0.110
Robot console time (min)	73.2 ± 22.6	81.4 ± 25.5	0.090
Perirenal fat dissection time (min)	10.5 ± 7.4	20.0 ± 10.8	<0.001
Warm ischemic time (min)	19.2 ± 8.7	19.5 ± 6.3	0.844
Intraoperative complication	1 (1.3%)	1 (2.6%)	0.587
Any postoperative complication	8 (10.0%)	4 (10.5%)	0.930
Estimated blood loss (cc)	102.2 ± 84.6	160.4 ± 287.7	0.325
Intraoperative transfusion	1 (1.3%)	3 (7.9%)	0.149
Postoperative transfusion	3 (3.8%)	3 (7.9%)	0.338
Negative surgical margin	79 (98.8%)	38 (100.0%)	0.489
Warm ischemic time ≤25 min	68 (85.0%)	34 (89.5%)	0.507
Post 1 year 90% GFR preservation rate	47 (60.3%)	26 (68.4%)	0.393
Post 1 year De Novo CKD ≥III rate	4 (5.2%)	5 (13.2%)	0.135
Hospital admission day	4.2 ± 1.2	4.8 ± 1.4	0.028

*MAP score, MAYO adhesive probability score; DM, Diabetes mellitus; HTN, Hypertension; CKD, Chronic kidney disease; ECOG, Eastern cooperative oncology group performance status; MDRD, Modification of Diet in Renal Disease; eGFR, estimated glomerular filtration rate; APF, Adherent perinephric fat*.

There were significant differences in age (*p* = 0.003), body mass index (BMI) (*p* < 0.001), presence of hypertension (*p* = 0.024), baseline serum creatinine (*p* = 0.004), and estimated glomerular filtration rate (*p* = 0.007) between the two groups (No APF vs. APF). The patients in the APF group have significantly higher MAP scores (*p* < 0.001), longer perirenal fat dissection time *(p* < 0.001), and longer hospital stay (*p* = 0.028). No significant differences are shown between groups in the rates of intraoperative or postoperative blood transfusion, warm ischemic time 25 min or less, intraoperative or postoperative complications, negative surgical margin rate, and 90% preservation of renal function at 1 year after surgery compared to preoperative period. The incidence of chronic kidney disease stage ≥3 at postoperative 1st year also did not differ significantly.

After multivariable logistic regression analyses (Table 2), MAP score (Odds ratio [OR]: 2.71, 95% Confidence interval [95% CI]: 1.56–4.71, *p* < 0.001), BMI (OR: 1.24, 95% CI: 1.04–1.47, *p* = 0.016), and perirenal fat dissection time (OR: 1.11, 95% CI: 1.03–1.19, *p* = 0.004) were identified to be significantly associated with APF.

**Table 2:** Associations of APF presence during robot-assisted partial nephrectomy (RAPN) with perioperative factors.

**Variable**	**Univariate analysis**			**Multivariate analysis**		
	**OR**	**95% CI**	***p*** **-value**	**OR**	**95% CI**	***p*** **-value**
Age	1.05	1.01–1.08	0.004	1.04	0.99–1.09	0.107
MAP score	2.85	1.87–4.34	<0.001	2.71	1.56–4.71	<0.001
Body mass index	1.22	1.08–1.37	0.001	1.24	1.04–1.47	0.016
Male gender	2.13	0.83–5.48	0.116			
ECOG PS	0.96	0.33–2.77	0.935			
DM	2.81	0.80–9.89	0.107			
HTN	2.48	1.11–5.51	0.026	0.48	0.14–1.66	0.249
Preoperative serum creatinine	13.06	1.99–85.69	0.007	2.08	0.15–28.07	0.358
RENAL score	1.02	0.83–1.26	0.869			
Tumor size	1.03	1.01–1.06	0.014	1.02	0.98–1.05	0.320
Intraoperative complication	2.14	0.13–35.08	0.595			
Any postoperative complication	1.06	0.30–3.76	0.930			
Length of hospital stay	1.40	1.03–1.90	0.031	1.08	0.71–1.65	0.723
Perirenal fat dissection time	1.12	1.06–1.18	<0.001	1.11	1.03–1.19	0.004

*OR, odds ratio; CI, Confidence interval; MAP score, MAYO adhesive probability score; DM, Diabetes mellitus; HTN, Hypertension; CKD, Chronic kidney disease; ECOG PS, Eastern cooperative oncology group performance status; APF, Adherent perinephric fat*.

Multivariate analyses (Table 3) in each surgeon subgroup for evaluating factors associated with prolongation of the perirenal fat dissection time revealed that perirenal fat dissection time was significantly correlated with APF in two surgeon subgroups (ß = 8.117, *p* = 0.023 for surgeon A subgroup, ß = 7.138, *p* = 0.058 for surgeon B subgroup, and ß =7.239, *p* = 0.011 for surgeon C subgroup).

**Table 3:** Linear regression analysis in each surgeon subgroup for evaluating factors associated with prolongation of the perirenal fat dissection time.

**Variable**	**Univariate analysis**			**Multivariate analysis**		
	**ß**	**95% CI**	***p*** **-value**	**ß**	**95% CI**	***p*** **-value**
**Surgeon A**						
Age	0.107	−0.113 to 0.327	0.333			
MAP score	2.310	0.543 to 4.076	0.012	0.268	−1.636 to 2.171	0.778
APF	12.308	6.868 to 17.747	<0.001	8.117	1.180 to 15.054	0.023
BMI	0.504	−0.237 to 1.245	0.178			
Female gender	−4.035	−10.352 to 2.282	0.205			
ECOG PS	12.128	2.235 to 22.021	0.017	6.596	−2.539 to 15.731	0.152
DM	−1.467	−16.033 to 13.100	0.840			
HTN	2.131	−3.957 to 8.220	0.484			
Serum Cr	19.908	8.468 to 31.349	0.001	10.541	−1.283 to 22.365	0.079
RENAL score	0.067	−1.318 to 1.452	0.923			
Tumor size	0.179	0.005 to 0.352	0.044	0.075	−0.085 to 0.234	0.187
**Surgeon B**						
Age	0.340	0.045 to 0.634	0.025	0.069	−0.230 to 0.368	0.581
MAP score	2.699	0.621 to 4.778	0.012	−0.137	−2.538 to 2.263	0.908
APF	10.745	3.771 to 17.718	0.003	7.138	−0.251 to 14.526	0.058
BMI	0.270	−0.730 to 1.270	0.587			
Female gender	−1.838	−9.419 to 5.744	0.626			
ECOG PS	−0.929	−11.162 to 9.304	0.855			
DM	1.000	−11.840 to 13.840	0.875			
HTN	10.169	3.889 to 16.448	0.002	6.131	−0.322 to 12.585	0.062
Serum Cr	−1.194	−18.834 to 16.445	0.892			
RENAL score	1.043	−0.529 to 2.615	0.187			
Tumor size	0.301	0.106 to 0.496	0.003	0.213	0.025 to 0.400	0.028
**Surgeon C**						
Age	0.147	−0.038 to 0.332	0.115			
MAP score	2.679	0.561 to 4.797	0.015	1.492	−0.594 to 3.578	0.154
APF	10.00	5.312 to 14.696	<0.001	7.239	1.763 to 12.715	0.011
BMI	0.640	−0.165 to 1.445	0.115			
Female Gender	−1.458	−8.345 to 5.428	0.668			
ECOG PS	3.678	−2.360 to 9.716	0.223			
DM	5.474	−1.913 to 12.861	0.141			
HTN	5.208	−0.783 to 11.199	0.086			
Serum Cr	6.381	−6.131 to 18.892	0.306			
RENAL score	1.109	−0.274 to 2.492	0.112			
Tumor size	0.154	0.009 to 0.317	0.043	0.114	−0.025 to 0.254	0.104

*APF, Adherent perinephric fat; CI, Confidence interval; MAP score, MAYO adhesive probability score; DM, Diabetes mellitus; HTN, Hypertension; ECOG PS, Eastern cooperative oncology group performance status; Cr, Creatinine*.

## Discussion

Adherent perinephric fat is characterized by inflammatory adipose tissues surrounding kidneys. It has earned substantial attention because of its overall effect on operative outcomes (6). It can be a demanding surgical factor by raising the surgical difficulty and causing it more urgent to identify and resect masses. Some investigations have reported the influence of APF on perioperative outcomes of PN (3, 4, 7). However, recently available classification systems to evaluate the complexity of PN are only based on tumor-specific factors such as location and size without considering patient-specific factors. The exact APF volume can be one of the evaluation tools for the complexity of RAPN (8–10). One meta-analysis (11) has revealed no significant impact of APF on the major complications' rate (*p* = 0.63) or the operative time (*p* = 0.08). However, estimated blood loss (EBL) was larger in the APF group (standardized mean difference [SMD]: 4, 95% CI:.22–.61, *p* = 0.0001). APF was also related to a longer operative time (SMD: 2.21, 95% CI:.02–4.41, *p* = 0.04) and an increased probability of conversion to open/ laparoscopic technique and radical nephrectomy (RR: 14.32, 95% CI: 2.91–70.50, *p* = 0.001). No significant difference was shown in EBL (*p* = 0.10). However, the transfusion rate was greater in the APF group (RR: 1.97, 95% CI: 1.13–3.43, *p* = 0.017). APF was not shown to be related to positive surgical margins (*p* = 0.64), major complications (*p* = 0.13), or rates of any complications (*p* = 0.87). One study (12) has reported that high MAP score (OR: 24.6, *p* < 0.001) and male factor (OR: 11.9, *p* < 0.01) were independent predictors of APF. Our findings are somewhat in line with those findings. Another study (13) has concluded that console time is significantly affected by gender, perinephric fat volume, and MAP score, with only perinephric fat volume showing an independent association with console time. In our study, there was no significant association between APF and total console time, although APF showed a significant association with perinephric fat dissection time. It was assumed that the reason for the no significant association between the two was because there were several experienced surgeons involved in the study and the total console time differed depending on the characteristics of the operator. Although not statistically significant, the longer operation time in the APF group was believed to be due to a higher RENAL score in the APF group. Other studies (14, 15) have shown that a higher RENAL score is associated with a longer operation time.

Unlike other studies based on single surgeons (1, 5, 16), our study had a strength in that it revealed significantly longer perinephric fat dissection time for patients with APF regardless of the number of surgeons, although the operation time and overall console time varied due to the nature of each operator. One study (17) has investigated patients undergoing laparoscopic PN and revealed that a higher MAP score is related to a longer operative time *(p* < 0.001) and a longer dissection time (*p* < 0.001). The EBL was increased in those with a higher MAP score (*p* < 0.001). No significant difference was shown in warm ischemic time (21 min vs. 20 min, *p* = 0.370) between the two MAP score groups. Male gender, BMI, and MAP score were significantly related to the prolongation of dissection time. Another study (18) has concluded that 49% have intraoperative detection of APF and that the MAP score has a distinguished ability to predict APF in open PN (AUC: 0.82, 95% CI: 0.74–0.92). The presence of APF was associated with higher EBL (*p* = 0.003) and longer operative times (*p* = 0.004). Although statistically insignificant, they suggested that APF might be associated with prolonged length of stay (>3 days) and postoperative complications. Another strength of the present study was that the presence of adherent perinephric fat was confirmed through a video review by a single experienced surgeon. One Asian cohort study (19) has revealed posterior perinephric fat thickness (PPFT), one component of MAP score, on preoperative CT and examined the relationship between techniques and surgical complexity in PN. For evaluation of PPFT, intraclass correlation coefficients between reviewers utilizing two detailed methods revealed insignificant differences (*p* = 0.173). It was shown as a determinant of operative time (*p* ≤ 0.023) in RAPN. Nevertheless, the presence of APF was not confirmed during surgeries in their study. The objectivity of APF presence could be increased as the existence of APF was not evaluated by only one surgeon in previous studies (1, 5, 16) and a third party confirmed it again through video review. In our study, multivariate analysis confirmed a significant association between BMI and APF, consistent with other previous studies (2, 17, 20). Khene et al. (2) have reported that hypertension (OR: 3.7, *p* = 0.02), higher BMI (OR: 1.2, *p* = 0.007), and male gender (OR: 13.2, *p* < 0.0001) are independent predictors of APF when the MAP score is not involved in the analysis. Yao et al. (17) have found that MAP score (b = 9.958, *p* = 0.002), male gender (b = 11.199, *p* = 0.001), and BMI (b = 1.197, *p* = 0.008) are significantly related to the prolongation of dissection time. Narita et al. (20) have also revealed that BMI (OR, 1.213; *p* = 0.013) is significantly associated with severe APF.

This study has several limitations, including its retrospective feature and single-institution design with a limited comparison group. In addition, our results might not be replicated in patients with a single kidney or clinical settings without a robotic surgical system as we only involved patients with bilateral kidneys who receive RAPN. One of the other limitations is that our center's surgical technique is different from other centers. Thus, caution is needed when applying the definition of perirenal fat dissection time. In our institution, after the surgeon started dissection of the renal hilum first with identifying the vessel, the surgeon then proceeded with the perirenal fat dissection. Therefore, the starting point of perinephric fat dissection time was determined after completing the vessel identification and secureness. Nevertheless, this corresponded to our purpose to elucidate whether the difficulty of the operation could be influenced by the time measuring only the moment for fat dissection around the tumor. Finally, the criteria for dividing patients into APF or no APF groups were still arbitrary even though we defined how APF can be detected. Notwithstanding these limitations, to the best of our knowledge, this is the first report evaluating perinephric fat as one of the factors determining the difficulty of robotic surgery in a cohort of pure Asians who underwent robotic surgery only.

## Conclusions

The MAP score and perirenal fat dissection time were significantly associated with APF during RAPN. Perioperative evaluation of technical difficulty can be evaluated with these factors. Our study suggests that these factors can act as reliable predictors of RAPN's complexity. To further investigate the significance of APF during RAPN, the impact of APF on surgical difficulty needs to be assessed with more diverse cohorts.

## Data Availability Statement

The raw data supporting the conclusions of this article will be made available by the authors, without undue reservation.

## Ethics Statement

The studies involving human participants were reviewed and approved by Institutional review board (IRB) of Seoul National University Bundang Hospital (NO. B-2108-702-102). Written informed consent for participation was not required for this study in accordance with the national legislation and the institutional requirements.

## Author Contributions

HK and SL: conception and design and revision of the manuscript. HK, MK, S-SB, SKH, and SL: data acquisition. HK, MK, and SL: analysis and interpretation of data and statistical analysis. HK: drafting of the manuscript. SL: supervision. All authors listed have made a substantial, direct and intellectual contribution to the work, and approved it for publication.

## Funding

This work was supported by the Korea Medical Device Development Fund grant funded by the Korea government (the Ministry of Science and ICT, the Ministry of Trade, Industry and Energy, the Ministry of Health & Welfare, the Ministry of Food and Drug Safety) (Project Number: 1711138269, KMDF_PR_20200901_0141) (NTIS, KMDF-RnD RS-2020-KD000141). This work was also supported by the National Research Foundation of Korea (NRF) grant funded by the Korea government (MSIT) (No. NRF-2020R1F1A1072702).

## Conflict of Interest

The authors declare that the research was conducted in the absence of any commercial or financial relationships that could be construed as a potential conflict of interest.

## Publisher's Note

All claims expressed in this article are solely those of the authors and do not necessarily represent those of their affiliated organizations, or those of the publisher, the editors and the reviewers. Any product that may be evaluated in this article, or claim that may be made by its manufacturer, is not guaranteed or endorsed by the publisher.
